# Synergistic eradication of *Candida albicans* prosthetic valve endocarditis with liposomal amphotericin B and high-dose caspofungin: a case report

**DOI:** 10.3389/fmed.2026.1704496

**Published:** 2026-03-04

**Authors:** Qing Feng, Lei Huang, Hua Luo, Xi Peng, Sheng Zhang

**Affiliations:** 1 Department of Intensive Care Unit (ICU), Peking University Shenzhen Hospital, Shenzhen, Guangdong, China; 2 Department of Dermatology, Peking University Shenzhen Hospital, Shenzhen, Guangdong, China

**Keywords:** antifungal agents, *Candida albicans*, echocardiography, fungal endocarditis, non-surgical treatment, prosthetic valve endocarditis, transesophageal echocardiography

## Abstract

Prosthetic valve endocarditis (PVE) due to *Candida albicans* was diagnosed in a 58-year-old woman 1 year after mitral bioprosthetic replacement for severe regurgitation. The diagnosis was prompted by recurrent fever and confirmed by positive blood cultures and echocardiographic vegetations. The isolate was susceptible to amphotericin B, azoles, and echinocandins. Despite meeting surgical criteria, the patient declined reoperation. Salvage therapy with liposomal amphotericin B combined with high-dose caspofungin successfully resolved symptoms and led to vegetation regression on follow-up imaging. The patient was discharged on lifelong suppressive antifungal therapy based on susceptibility profiling.

## Introduction

*Candida albicans (C. albicans) is* a commensal fungus commonly colonizing the human oral cavity, gastrointestinal tract, and vagina, typically existing in equilibrium with the normal flora. However, under conditions such as immunosuppression, prolonged broad-spectrum antibiotic or immunosuppressive therapy, and nosocomial exposure, *C. albicans* can proliferate and cause infection (1). It is known to be a rare cause of infective endocarditis (IE). The prevalence of Fungal IE is between 1 and 10% of all IE cases (2). The incidence of fungal endocarditis or IE has been increasing due to the frequent use of external devices, ranging from prosthetic heart valves to central venous catheters, and it has a poor prognosis, with mortality greater than 50% (3–5). *Candida* and *Aspergillus* species are the predominant fungal pathogens responsible for Fungal IE, Among Candida endocarditis, *C. albicans* is the main cause of Fungal IE (6).

Here, we report a case of *C. albicans* Prosthetic valve endocarditis (PVE) occurring 1 year after mitral valve bioprosthesis replacement in a 58-year-old woman. The patient presented with recurrent fever and was diagnosed with *C. albicans* endocarditis through blood cultures and echocardiography. Despite meeting surgical indications, the patient declined reoperation. She was successfully treated with a combination of Amphotericin B liposomes and high-dose caspofungin. New vegetations on the patient’s prosthetic mitral valve disappeared, following this combinatorial therapy for 45 days. This case report highlights the virulence of *C. albicans* in PVE along with the diagnostic and therapeutic challenges inherent in fungal endocarditis. It also emphasizes the importance of early detection, appropriate antifungal therapy, and multidisciplinary management required to enhance clinical awareness of this entity.

## Case presentation

We present the case of a 58-year-old woman readmitted to the Intensive Care Unit (ICU), Peking University, Shenzhen Hospital on August 6, 2024, with recurrent fever (a maximum temperature of 39 °C was recorded), persisting for nearly 2 weeks. The patient had no history of long-term hormone use, total parenteral nutrition, or malignancies, with no significant personal history of smoking or alcohol abuse. Initial blood count showed White Blood Cells (WBC) count of 17.18 × 10^9^ cells/L (79.5% neutrophils, 16.3% lymphocytes). Procalcitonin (PCT), troponin T, N-terminal pro-B-type natriuretic peptide (NT-proBNP) and (1,3)-β-D glucan were elevated 6.81 ng/mL, 0.161 ng/mL, 4,679 pg./mL and 663.70 pg./mL, respectively. The combination of high-grade fever and a significantly elevated (1,3)-β-D glucan level was highly suggestive of a fungal infection. We initiated empirical antifungal therapy with fluconazole (loading dose 400 mg IV twice, followed by a maintenance dose: 200 mg every 12 h). On Day 3, blood culture grew yeast-like organisms identified as *C. albicans*. Antifungal susceptibility testing confirmed sensitivity to amphotericin B, voriconazole, fluconazole, caspofungin, micafungin, and anidulafungin (Table 1). The Transthoracic Echocardiography (TTE) showed thickened and coarse valve leaflets with a mobile echo density (approximately 25 × 6 mm) on the anterior leaflet, suggesting recurrent IE with a new vegetation. The left heart remained mildly enlarged, and left ventricular ejection fraction (LVEF) was preserved (50%). A diagnosis of *C. albicans* PVE was established. Then, we started antifungal therapy with caspofungin (loading dose 70 mg IV, then 50 mg IV daily).

**Table 1:** Antifungal drug sensitivity tests to *C. albicans.*

Antifungal drug	Sensitivity	Result
Amphotericin B	S	0.5
Fluconazole	S	0.5
Itraconazole		0.06
Voriconazole	S	≤0.008
Flucytosine		≤0.06
Caspofungin	S	0.06
Micafungin	S	≤0.008
Posaconazole	WT	0.015
Anidulafungin	S	0.03

S, sensitive; WT, wild type.

On day 9, the patient was referred to the Cardiac Surgery department for evaluation of surgical timing. However, subsequent blood cultures remained positive for *Candida albicans* (1,3)-β-D-glucan levels remained elevated (510.00 pg./mL), and the patient’s temperature exhibited an upward trend, fluctuating between 38.5 °C and 39.3 °C. On Day 13, the cardiothoracic surgeon initiated combination antifungal therapy with caspofungin (50 mg IV once daily) and voriconazole (loading dose 400 mg IV for the first two doses, followed by 200 mg IV twice daily). Despite these adjustments, the patient’s temperature fluctuated between 36.5 °C and 37.7 °C. However, blood cultures persistently grew *C. albicans* with a concomitant increase in (1,3)-β-D-glucan levels to 924.44 pg./mL, with the patient’s condition deteriorating rapidly. On Day 24, the patient on presenting with acute heart failure and pulmonary edema, was subsequently transferred to the ICU for further treatment. Bedside echocardiography showed thickened bioprosthetic mitral valve leaflets, with a small strip (approximately 8 × 6 mm) fluttering on the anterior leaflet, and a decreased LVEF (44%).

Given the progression of fungal endocarditis, we modified antifungal therapy on Day 25 to caspofungin (50 mg IV once daily) combined with amphotericin B cholesteryl sulfate complex colloidal dispersion (ABCD) (7, 8). To mitigate potential adverse effects, we administered ABCD in an ascending dose regimen for three consecutive days: 50, 100, and 150 mg. Despite premedication with dexamethasone and antihistamines, as well as absence of hypokalemia (Potassium levels 3.8–4.2 mmol/L) the patient developed severe malignant arrhythmias on day 3 of ABCD therapy, characterized by frequent premature ventricular contractions, atrial fibrillation, and short runs of ventricular tachycardia. The electrocardiogram (ECG) is available in the Supplementary Figure S1. Based on the temporal correlation between the onset of arrhythmia and medication administration, we suspected a high likelihood of ABCD-induced cardiotoxicity. Consequently, we administered intravenous lidocaine to manage the arrhythmia and immediately discontinued ABCD. On Day 28, we modified the antifungal regimen to a combination therapy consisting of liposomal amphotericin B (L-AmB, 250 mg IV once daily) and caspofungin (50 mg IV once daily).

**Figure 1:**
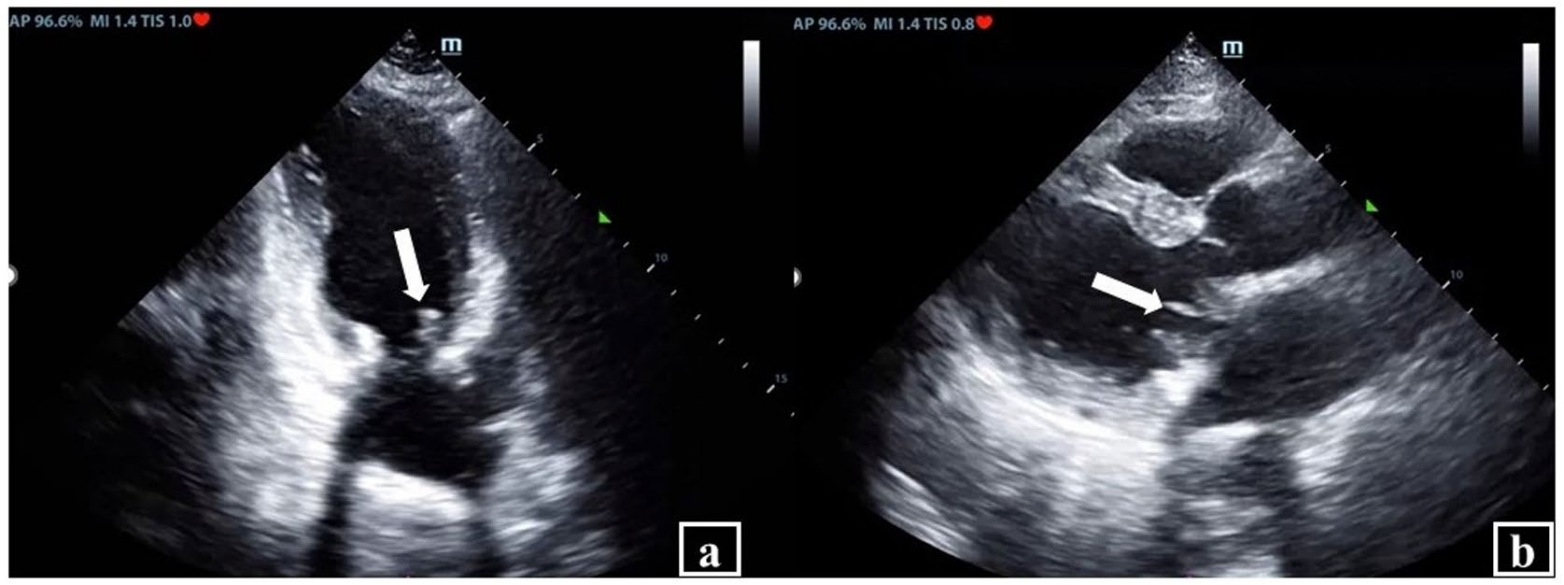
Transthoracic echocardiography (TTE) in *Candida albicans* complex prosthetic valve endocarditis. **(a)** TTE demonstrated thickened mitral bioprosthetic valve leaflets, a 9 × 5 mm flocculent (arrow). **(b)** Accompanied by restricted valve opening and incomplete closure (arrow).

During this period, we conducted comprehensive discussions with the patient’s family regarding the indications and risks of surgical re-intervention, they ultimately opted for conservative medical management. Following the adjustment of the therapeutic regimen, the malignant arrhythmias resolved successfully. However, the patient continued to exhibit persistent fever (temperature range: 36.1 °C–37.7 °C), elevated (1,3)-β-D-glucan levels of 865.18 pg./mL, and serial blood cultures remained positive for *C. albicans* On Day 36, we intensified the antifungal regimen by increasing the caspofungin dosage to 100 mg IV once daily in combination with L-AmB (250 mg IV once daily), with rigorous monitoring of hepatic and renal function. This intervention resulted in normalization of the patient’s body temperature on Day 37, accompanied by negative blood cultures. TTE demonstrated thickened mitral bioprosthetic valve leaflets, a 9 × 5 mm flocculent, accompanied by restricted valve opening and incomplete closure (Figure 1).

**Figure 2:**
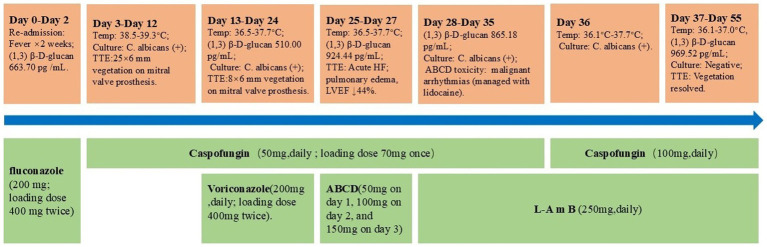
Timeline of the antifungal therapies administered and clinical events during hospitalization.

On Day 51, TTE reexamination revealed complete resolution of the previously visualized vegetations, the prosthetic valve demonstrated normal leaflet mobility and hemodynamic function, LVEF improved to 58%. However, serum (1,3)-β-D-glucan levels remained significantly elevated at 969.52 pg./mL. The patient was discharged on Day 55 and transferred to a local healthcare facility with recommendations for lifelong suppressive antifungal therapy guided by susceptibility results. Regular follow-up with echocardiography and blood cultures was strongly advised. Timeline of the antifungal therapies and clinical events during hospitalization is described in Figure 2.

**Figure 3:**
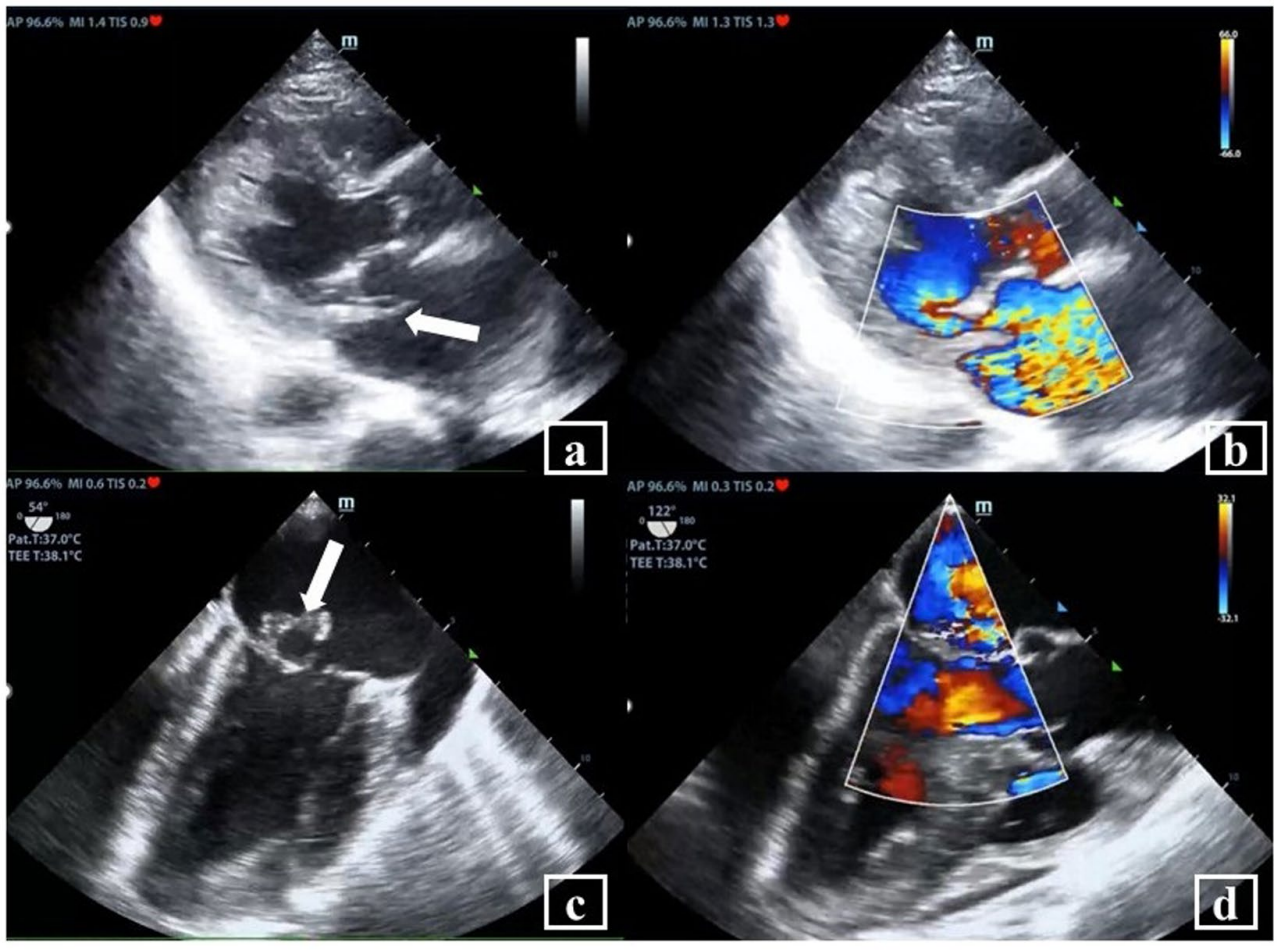
Transthoracic echocardiography (TTE) and transesophageal echocardiography (TEE). **(a)** TTE showed posterior mitral valve tendon cord rupture and posterior mitral valve prolapse (MVP) (arrow); **(b)** severe mitral regurgitation (MR) was observed on Doppler color view (TTE); **(c)** TEE showed a vegetation on the mitral valve and the vegetation was about 13 × 4.0 mm (arrow); **(d)** severe eccentric regurgitation signal of the mitral regurgitation (MR) was observed on Doppler color view (TEE).

**Figure 4:**
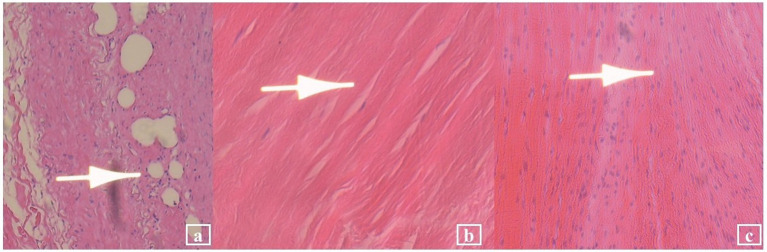
Histopathological examination (HPE) results (HE staining; 100×) showed: **(a)** Small vessel hyperplasia (arrow); **(b)** interstitial hyalinization (arrow); and **(c)** abundant collagen fibers (arrow).

The current episode of Fungal IE PVE occurred in the context of a complex medical history. One year prior to this admission (April 2023), the patient was initially admitted to the ICU due to dyspnea persisting for 1 month. TTE performed showed a ruptured posterior mitral valve tendon cord, and a vegetation was present. TEE confirmed the infective endocarditis with vegetations (Figure 3). Vasculitis serology testing demonstrated positivity for two markers: Proteinase 3—Anti-Neutrophil Cytoplasmic Antibody (PR3-ANCA) (+++), Cytoplasmic Anti-Neutrophil Cytoplasmic Antibody (cANCA) (+). The patient also exhibited multi-organ/ system damage that included anemia, acute renal and respiratory failure. Metagenomic next-generation sequencing (mNGS) of the blood performed after negative blood cultures, revealed the presence of *Streptococcus* species. She was managed with antibiotics [ceftriaxone (2 g IV twice daily) and ampicillin-sulbactam (1.5 g IV four times daily)]. After aggressive management of infection and heart failure, the patient’s IE and heart failure stabilized. Her vasculitis improved with pulse steroid therapy, plasma exchange, and cyclophosphamide immunosuppression, accompanied by improvement in renal function and coagulation parameters. She underwent mitral valve bioprosthesis replacement, tricuspid valvuloplasty, and temporary cardiac pacemaker implantation 1 month later. Postoperative histopathological examination (HPE) of the excised mitral valve revealed sallow grayish-brown tissue with focal small vessel hyperplasia, interstitial hyalinization, and abundant collagen fibers, with no pathogen being detected (Figure 4). The intensified antibacterial regimen was maintained throughout the patient’s first hospitalization. During this period, the body temperature fluctuated within the normal range (36.2 °C–36.9 °C), with all follow-up blood cultures returning negative. The patient was discharged two and a half months later, with instructions to continue antimicrobial therapy based on susceptibility results. Notably, during the current admission, the patient’s vasculitis-related antibodies PR3-ANCA and cANCA were negative.

The antifungal regimen was intensified during follow-up, with the oral fluconazole dose titrated according to the patient’s creatinine clearance, the serum (1,3)-β-D-glucan levels declined from 162.55 pg./mL (on day 60) to 80.29 pg./mL (on day 140) and subsequently normalized, with consistently negative monthly blood cultures and echocardiograms.

## Discussion

Fungal IE is characterized by direct fungal invasion of the cardiac endothelium, primarily involving valves, mural endocardium, or adjacent great vessels, resulting in vegetation formation. The relatively rare occurrence of fungal IE, combined with its nonspecific clinical manifestations and diagnostic challenges, demands a heightened clinical suspicion, particularly in patients with established risk factors such as prosthetic heart valves and prior IE (1, 2, 7, 8). The incidence of fungal IE has risen in recent decades primarily due to expanded utilization of invasive medical devices and procedures (9). Other significant predisposing factors include underlying structural heart disease, intravenous drug use, immunosuppression, prolonged broad-spectrum antibiotic exposure, and chronic comorbidities (1, 9). In this case, the patient developed *C. albicans* PVE 1 year after mitral valve replacement, demonstrating the persistent infection risk associated with prosthetic materials. This patient’s history of IE, invasive procedure and long-term antibiotic exposure likely contributed to her susceptibility to *C. albicans* endocarditis. Moreover, her early occurrence of multi-organ involvement may have exacerbated her immunocompromised state.

Establishing a diagnosis of fungal IE is inherently difficult due to nonspecific clinical features. The Modified Duke Criteria, the widely accepted diagnostic framework for IE (5, 10), were primarily derived from bacterial IE data and lack specificity for fungal IE. Blood cultures, while often the initial diagnostic step, exhibit low sensitivity, especially early in the infection course or following partial antifungal treatment (1, 3). In cases of *Candida endocarditis*, diagnostic sensitivity may be moderately elevated, autopsy studies demonstrate positive blood cultures in 50–100% of confirmed cases. Enhanced sensitivity can be achieved by collection of serial blood cultures and/or increasing blood culture volumes (6, 11). In the present case, persistently positive blood cultures for *C. albicans* were pivotal in confirming the diagnosis. However, echocardiographic visualization of new vegetations on the prosthetic mitral valve was equally essential. TTE and TEE are indispensable imaging modalities for identifying valvular abnormalities and vegetations, although differentiating infectious vegetations from sterile thrombi can be challenging (12).

The (1,3)-β-D-glucan assay is a non-invasive test that works by detecting a specific polysaccharide (1,3)-β-D-glucan in the fungal cell wall, and it is commonly used in the diagnosis of invasive fungal diseases (13). At a cutoff of 60 pg./mL, it demonstrates a sensitivity of 69.9% and a specificity of 87.1% in candidemia, with positive and negative predictive values of 83.8 and 75.1%, respectively (14, 15). Elevated (1,3)-β-D-glucan levels suggest invasive fungal infection. Despite clinical improvement and negative blood cultures following combination therapy, the patient’s (1,3)-β-D-glucan levels remained elevated at discharge (969.52 pg./mL), likely reflecting persistent fungal antigen load or biofilm-associated infection rather than ongoing fungemia. During oral fluconazole therapy, the (1,3)-β-D-glucan levels declined from 162.55 pg./mL (day 60) to 80.29 pg./mL (day 140), underscoring its value as a prognostic marker when interpreted within the full clinical context.

Despite advances in diagnostics and newer antifungals, mortality associated with fungal IE remains unacceptably high (7, 16). Management of fungal IE typically necessitates a dual approach: prolonged, targeted antifungal therapy combined with surgical intervention when feasible. A multidisciplinary team comprising specialists in cardiology, cardiothoracic surgery, infectious diseases, and pharmacy is essential (2, 5). A systematic review encompassing 250 fungal IE patients strongly advocated for combined surgical and antifungal therapy to improve outcomes (1). In this case, initial monotherapy with caspofungin failed to achieve microbiological clearance, and clinical deterioration ensued, prompting sequential therapeutic modifications. The subsequent combination of voriconazole and caspofungin yielded partial clinical improvement but failed to eradicate the fungemia. Antifungal selection must be guided by susceptibility testing. Recent trends indicate a preference for combination antifungal therapy and increased echinocandin use (7, 17, 18). As recommended in the IDSA 2016 guidelines, initial regimens include L-AmB (3–5 mg/kg daily) with or without flucytosine (25 mg/kg four times daily), or high-dose echinocandins (caspofungin 150 mg daily, micafungin 150 mg daily, or anidulafungin 200 mg daily) (7, 8). While adding flucytosine to AmB reduces mortality for *Candida* spp., its utility is limited by dose-dependent bone marrow toxicity (19, 20). Echinocandins exert potent fungicidal activity against most *Candida species*, primarily due to their superior penetration into biofilms (21). This effect is complemented by L-AmB, which demonstrate enhanced efficacy against biofilms on prosthetic devices (1). Clinical and experimental evidence supports combination therapy efficacy. In murine systemic candidiasis models, caspofungin combined with L-AmB achieved greater renal fungal burden reduction than monotherapy (22). Similarly, L-AmB plus an echinocandin significantly improved survival in murine systemic *C. glabrata* infection (23). This strategy is clinically relevant: a preterm neonate with *Candida conglobata* bloodstream infection refractory to L-AmB monotherapy was successfully treated with a regimen including caspofungin (24). In this patient, L-AmB combined with high-dose caspofungin ultimately proved successful, resulting in defervescence and clearance of fungemia (7, 8, 25–27). The isolate demonstrated susceptibility to multiple antifungals, including amphotericin B, voriconazole, fluconazole, caspofungin, and micafungin. However, clinical response and potential adverse effects are critical considerations. The patient experienced severe, treatment-limiting malignant arrhythmias attributed to ABCD, necessitating a switch to the better-tolerated L-AmB formulation, underscoring the importance of therapeutic drug monitoring and formulation selection (27, 28). Surgical intervention is frequently indicated in fungal IE, particularly for persistent infection despite optimal medical therapy, valve dysfunction, heart failure, or embolic complications (29, 30). In this case, the patient and family declined reoperation due to perceived risks. Two clinical cases of prosthetic valve-associated *Candida parapsilosis* endovascular infections that resolved with combination antifungal therapy, avoiding surgical intervention have been reported previously (19). These reports underscore the importance of comprehensive risk–benefit assessment and shared decision-making in complex clinical scenarios. Managing fungal IE poses formidable challenges, successful management typically requires a multifaceted approach combining prolonged antifungal therapy, prompt surgical intervention for source control.

A previously reported case illustrated a severe instance of *C. albicans* prosthetic valve endocarditis in a young adult with multiple risk factors, including a complex cardiac history and acute renal failure. Successful management of this case involved homograft valve replacement, targeted antifungal therapy, and vascular surgery for complications, ultimately leading to a positive outcome with suppressive fluconazole upon discharge (31). This case illustrates the challenges in microbiological eradication, evidenced by persistent positive blood cultures despite rigorous antifungal regimens. Significant drug-related toxicities, such as ABCD-induced cardiotoxicity, further complicates management. Another challenge is the necessity for prolonged, often lifelong, antifungal therapy with strict adherence. Fungal IE harbors a high recurrence rate even after combined medical treatment and surgical intervention, mandating long-term suppressive therapy (32, 33). As per IDSA 2016 guidelines, for patients who cannot undergo valve replacement, long-term suppression with fluconazole, 400–800 mg (6–12 mg/kg) daily, if the isolate is susceptible, is recommended (7). This patient was discharged on indefinite antifungal suppression, highlighting the mandatory requirement for comprehensive patient and family education, regarding the imperative of sustained therapy and vigilant monitoring. Non-adherence significantly increases the relapse risk in patients (34, 35). Additionally, clinicians must maintain vigilance for less common pathogens like *Candida parapsilosis* PVE, as early diagnosis and prompt therapy may improve outcomes (36). While combined surgical resection and antifungal therapy remains the gold standard, managing higher surgical risk patients with retained prostheses presents unique challenges, including breakthrough infections, complex pharmacokinetics, and cumulative toxicities from prolonged suppressive regimens. The strengths of our approach include rigorous serial monitoring of microbiological and inflammatory markers, timely antifungal escalation based on susceptibility profiles, and a multidisciplinary team decision-making process. However, the findings are limited by its single-center, non-controlled design. Further multicenter studies are warranted to validate the comparative efficacy of pharmacological interventions versus combined approaches.

From the patient’s perspective, she declined reoperation after weighing the risks against the benefits, preferring medical management. This preference was incorporated into a shared decision-making process. She has since adhered to lifelong fluconazole suppression therapy, with follow-up echocardiograms and blood cultures confirming sustained remission. This case highlights how aligning treatment with patient values, supported by consistent monitoring, can lead to successful outcomes.

## Conclusion

Fungal IE is a rare but devastating complication associated with significant mortality. This case report illustrates the diagnostic and therapeutic complexities of *C. albicans* PVE. Multidisciplinary treatment including tailored antifungal therapy and often surgery is essential. Shared decision-making on surgery timing and long-term anti-fungal therapy is critical. Our experience demonstrates combination therapy (L-AmB plus high-dose caspofungin) is a viable strategy, offering guidance for similar cases. Future research should optimize antifungal regimens and surgical strategies to improve outcomes.

## Data Availability

The original contributions presented in the study are included in the article/Supplementary material, further inquiries can be directed to the corresponding author.
