# Supplementary figures and images for: Synergistic eradication of Candida albicans prosthetic valve endocarditis with liposomal amphotericin B and high-dose caspofungin: a case report
Source: Front Med (Lausanne). 2026 Mar 4;13:1704496. doi: 10.3389/fmed.2026.1704496 (PMC12995660; doi:10.3389/fmed.2026.1704496)

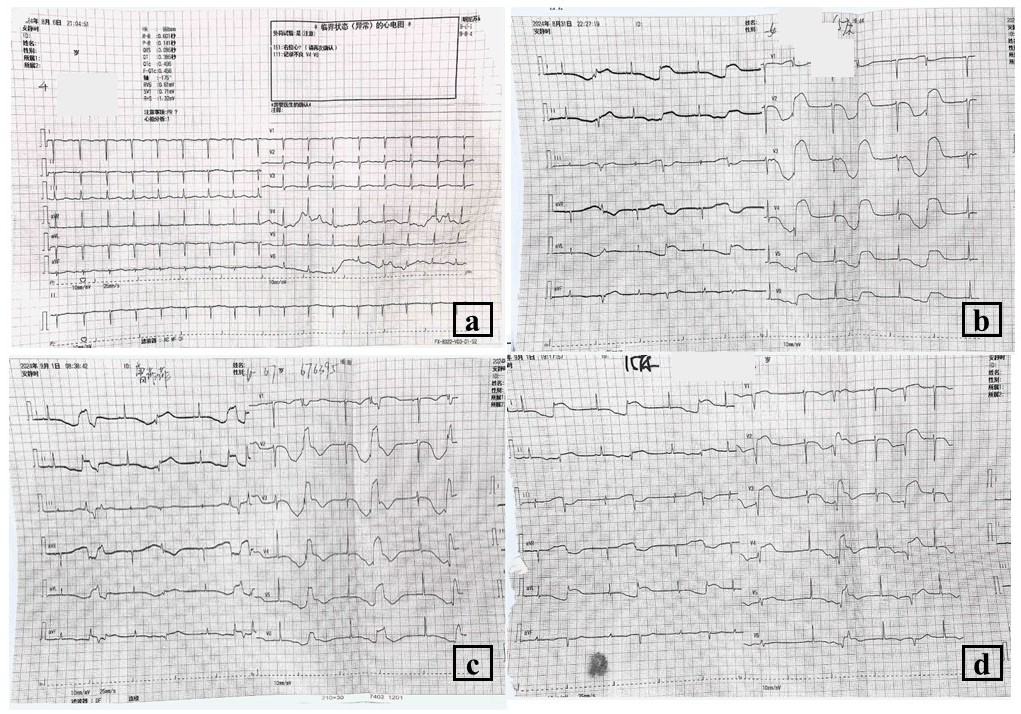

Supplement: Supplementary file 1 [file Image_1.jpg]
